# Out-Patients at St. Bartholomew's

**Published:** 1889-09-07

**Authors:** 


### The Hospital Workshop.

### IX.—OUT-PATIENTS AT ST. BARTHOLOMEW'S.

(BY OUR SPECIAL COMMISSIONER.)

So far as out-patients are concerned, St. Bartholomew's is the most frequented hospital in town, a fact abundantly proved by the following figures taken for the year 1887. Out-patients:

| | | |
|---|---|---|
| St. Bartholomew's. | 150,828 | (including casualties). |
| London ............... | 95,760 | ,, ,, |
| St. Thomas's ...... | 87,601 | ,, ,, |
| University College. | 44,382 | ,, ,, |
| Guy's ................. | 37,865 | ,, ,, |

It is seldom easy to make out exactly the reasons that guide and govern the populace. Here, however, their liking seems to be due to a number of different causes working together. 1. Patients receive attention quickly and at convenient hours. They come at nine o'clock in the morning, and are seen by members of the assistant staff. Slight cases have immediate treatment, while the more serious are directed to come again at 12.30 p.m. 2. Central position of the hospital, with no other great institution of the kind in the near neighbourhood. 3. Popularity of staff. 4. Rapidity of dispensing. 5. Freedom of admission. 6. The old-established reputation of the charity, which was founded in 1122. Sir James Paget lately pointed out that the first King Henry granted their original charter, and that the present one was given by the last King Henry.

An attempt is made to prevent abuse of the out-patient department by well-to-do people. In a letter which recently appeared in the *British Medical Journal*, Dr. Rentoul writes: "The St. Bartholomew's Hospital System.—Here, also, is an enquiry officer, who is paid £156 yearly. The secretary says he 'draws the patients' attention to the fact that the hospital is a charitable institution, designed for the relief only of the necessitous poor.' In 1882, the year previous to the appointment of an inspector, the number of casualty and out-patients was 170,030, while last year the total number was 144,681; a falling off of 25,349. Compare this plan with the very doubtful one pursued at Guy's Hospital, where they charge the sick poor threepence for advice and medicine. Who introduced this iniquitous system of a medical charity actually selling its charity?"

At 12.30 p.m. your commissioner, under the guidance of a student, went to the waiting-room for the surgical cases sent in from the nine o'clock inspection. In this place the "dressers" take careful notes of patients before passing them in for consultation. On entering, Mr. Butlin, the surgeon for the day, had before him a man with a swollen ankle-joint. The students were being questioned, one at a time, as to history, treatment, and symptoms, a reason being expected for every answer given. In this way, which is called "quizzing" by the Americans, the utmost is made of material from an educational point of view.

Crossing over to the medical branch, we enter a light and well-ventilated room where patients are seated waiting their turn for advice. At the particular moment of our arrival a white-faced girl with a smart bonnet is showing her tongue to her next-door neighbour. Without any great effort of imagination it may be guessed that she is suffering from what the doctors call "anæmia," or poverty of blood, and that she is taking iron, a remedy which is apt to blacken the tongue. Let us look at her card. She is nineteen years of age, a tailoress from Ludgate-hill. When admitted she complained of pains all over the body—in chest, in heart, and in joints. Breath short; easily fatigued; has been in this condition for two years. The mucous membrane of conjunctiva (*i.e.*, inside of eyelid), and also of lips, is pale and bloodless. Feet and legs sometimes swell in the morning. Heart pretty sound. The disease is entered as "morbid anæmia," and the medicine is citrate of iron and ammonia, a mild, blood-forming tonic. Under a course of prolonged treatment no doubt the girl's health will be very much improved; but good food and fresh air are almost as important as physic in these cases. "Anæmia" is a common disorder amongst those who have to spend their days in crowded workshops, and whose meagre earnings are often swallowed up by the demands of home. As a result, underfeeding is added to the burden of overwork and unhealthy surroundings, and the hospitals are crowded by these pale victims of a cruel industrial warfare.

In the consulting-room several persons are under examination. One is a dock-labourer from Poplar. He is fifty-nine years of age, and is sitting with hand pressed across the pit of his stomach. There is a somewhat anxious expression upon his weather-beaten face. Of late he has been losing flesh rapidly, and complains that he can no longer swallow solid food. There has been no pain, neither has he spat blood at any time, although he now brings up a quantity of watery fluid. The chest is barrel-shaped, owing to enlargement of the lungs from distension of the air-cells. Dr. Habershon's notes describe the patient as "a tall, spare man, with well-marked emphysema" (the state of lung mentioned above). Unhappily, there can be no doubt this case is one of malignant—that is to say, cancerous—disease of the stomach, which will run a rapid course, and against which treatment will be of little avail.

The arrangements for dispensing, an important part of the out-patient department, seem to be especially good. Most of the preparations are made in the compounding-room, excepting a few, such as the alkaloids and green extracts, which are bought along with the chemicals. By this means purity is ensured, as well as a great saving effected to the funds of the hospital. Without attempting anything like an elaborate description, some few points of interest may be noted for the behoof of readers. Near the entrance, worked round and round at high speed by steam power, are two millstones for grinding various crude drugs, such as rhubarb, senna, jalap, and bark, into powders. Hard by is a sifting-machine, which consists of a sieve fixed in a barrel, the whole being thoroughly shaken up by means of a simple yet ingenious eccentric motion. Other advanced appliances of the modern chemical laboratory are to be seen on every side. Here, for instance, enclosed in steam-jacket pans, are two basins for making the acid preparations. They are of platinum, the only metal capable of resisting the action of acids for any length of time; and must be very costly, not less than a couple of hundred pounds apiece at a rough guess. Their small size contrasts sharply with the huge mortar standing close by, and which looks as if it had strayed from the kitchen of some pantomime giant. His club might well be the pestle, a beam some three feet long, and worked in eccentric movement at the rate of 36 revolutions a minute. Nor is the provision of drugs on a less gargantuan scale. Sacks of Tinivelly senna, of gentian and of cinchona bark lie heaped on one side; here is a bin, high as a man's chest, filled with crushed poppy capsules. The latter are used chiefly for preparing syrup of poppies, that famous old remedy for cough mixtures. The dispenser tells us this bin is emptied eight times in the six months. The next compartment, of equal size, is for linseed meal, of which half a ton is consumed every two or three weeks, less being ordered in summer than in winter. Other crude drugs are stored up in various bins and crates; treacle is kept in large quantity for grinding up with powders into pill-mass. Down the room is ranged a row of twelve gallon jars, reminding one irresistibly of Ali Baba and the forty thieves. Over yonder are two big tanks—one of which holds olive and the other cod-liver oil. This line of casks contains the commoner medicines of the hospital pharmacopœia, made up ready for dispensing. Each of them, when filled, will last from one to three days. But not less than eighteen gallons daily are wanted of a quassia and iron mixture, and a similar quantity of a saline draught, known as "house mixture." Water is proverbially the right hand of the dispenser, and the wholesale production of physic that goes on here requires an apparatus capable of distilling some seventy gallons in a day.

On one side of the spirit-room are four pipes of wine, three of port, and the other of sherry. Opposite stand a row of tanks, one for whisky, another of a hundred galllon capacity for brandy, and a third twice that size for proof alcohol.

A few facts and figures of this kind will bring home to the reader an idea of the vastness of a charity which dispenses medicines in such titanic fashion. Dispensing is a very important part of the hospital machinery, and on its accuracy and the purity of its drugs must in a large measure depend the success of medical treatment. It is a department requiring no less energy in administration than constancy of supervision to ensure proper working. From what we have personally seen, St. Bartholomew's will bear favourable comparison in these respects with any of the great medical charities.

